# Internal Responsiveness of EQ-5D-5L and EORTC QLQ-C30 in Dutch Breast Cancer Patients during the First Year Post-Surgery: A Longitudinal Cohort Study

**DOI:** 10.3390/cancers16111952

**Published:** 2024-05-21

**Authors:** Noëlle J. M. C. Vrancken Peeters, Janine A. van Til, Anouk S. Huberts, Sabine Siesling, Olga Husson, Linetta B. Koppert

**Affiliations:** 1 Department of Surgical Oncology, Erasmus MC, University Medical Center Rotterdam, 3015 GD Rotterdam, The Netherlands; 2 Department of Medical Oncology, Netherlands Cancer Institute-Antoni van Leeuwenhoek, 1066 CX Amsterdam, The Netherlands; 3 Department of Health Technology and Services Research, Technical Medical Centre, University of Twente, 7522 NB Enschede, The Netherlands; 4 Department of Quality and Patient Care, Erasmus MC, University Medical Center Rotterdam, 3015 GD Rotterdam, The Netherlands; 5 Department of Research and Development, Netherlands Comprehensive Cancer Organisation (IKNL), 3511 DT Utrecht, The Netherlands

**Keywords:** Breast Cancer, European Organization for Research and Treatment of Cancer Quality of Life Questionnaire Core-30 (EORTC QLQ-C30), EuroQoL 5-Dimension 5-Level questionnaire (EQ-5D-5L), Patient-Reported Outcome Measures (PROMs), Responsiveness

## Abstract

**Simple Summary:**

Breast cancer is one of the most diagnosed malignancies among women worldwide, affecting 18,000 women annually in the Netherlands. As life expectancy increases, monitoring quality of life (QoL) using Patient-Reported Outcome Measures (PROMs) becomes more important. The EQ-5D-5L and the EORTC QLQ-C30 are two widely used PROMs in the Netherlands. For utilizing PROMs in research and clinical care, internal responsiveness is key. Given the broad application of these PROMs in the Netherlands and the fact that internal responsiveness varies based on the disease, treatment regime, and geographical factors, this study assesses and compares the internal responsiveness of the EQ-5D-5L and the EORTC QLQ-C30 among Dutch breast cancer patients during the first year post-surgery. The results demonstrated that the EQ-5D-5L and the EORTC QLQ-C30 have small internal responsiveness (<0.5) at 6- and 12 months post-surgery. These findings provide crucial insights for interpreting outcomes from both PROMs in research and clinical practice.

**Abstract:**

The EuroQoL 5-Dimension 5-Level questionnaire (EQ-5D-5L) and the European Organization for Research and Treatment of Cancer Quality of Life Questionnaire Core-30 (EORTC QLQ-C30) are commonly used Patient-Reported Outcome Measures (PROMs) for breast cancer. This study assesses and compares the internal responsiveness of the EQ-5D-5L and EORTC QLQ-C30 in Dutch breast cancer patients during the first year post-surgery. Women diagnosed with breast cancer who completed the EQ-5D-5L and EORTC QLQ-C30 pre-operatively (T0), 6 months (T6), and 12 months post-surgery (T12) were included. Mean differences of the EQ-5D-5L and EORTC QLQ-C30 between baseline and 6 months (delta 1) and between baseline and 12 months post-surgery (delta 2) were calculated and compared against the respective minimal clinically important differences (MCIDs) of 0.08 and 5. Internal responsiveness was assessed using effect sizes (ES) and standardized response means (SRM) for both deltas. In total, 333 breast cancer patients were included. Delta 1 and delta 2 for the EQ-5D-5L index and most scales of the EORTC QLQ-C30 were below the MCID. The internal responsiveness for both PROMs was small (ES and SRM < 0.5), with greater internal responsiveness for delta 1 compared to delta 2. The EQ-5D-5L index showed greater internal responsiveness than the EORTC QLQ-C30 Global Quality of Life scale and summary score. These findings are valuable for the interpretation of both PROMs in Dutch breast cancer research and clinical care.

## 1. Introduction

Breast cancer is one of the most commonly diagnosed malignancies among women worldwide [1,2]. Every year, about 18,000 women are diagnosed with breast cancer in the Netherlands and the incidence of invasive breast cancer is still increasing [3,4,5]. However, advancements in breast cancer treatments and early detection through nationwide screening programs have led to better prognoses for patients. While this improved prognosis is encouraging, breast cancer survivors may experience late-effect symptoms after diagnosis and completion of treatment, which can negatively affect their health status and health-related quality of life (HRQoL). Therefore, monitoring the health status and HRQoL of breast cancer patients throughout their disease trajectory and thereafter is important [5,6,7,8,9,10].

Patient-reported outcome measures (PROMs) are validated questionnaires that are used to measure HRQoL, health, and functional status of patients. They can be either generic or more disease-specific in nature [11,12]. Using PROMs in research and clinical care enables the monitoring of quality of care at a hospital level and provides clinicians with valuable information to better address the needs of each individual patient [13]. The EuroQoL 5-Dimension 5-Level questionnaire (EQ-5D-5L) and the European Organization for Research and Treatment of Cancer Quality of Life Questionnaire Core-30 (EORTC QLQ-C30) are commonly used PROMs in research and routine care for breast cancer patients in the Netherlands [14,15,16].

The EQ-5D-5L is a validated and standardized generic preference-based questionnaire that measures a patient’s health status using five dimensions: mobility, self-care, usual activities, pain/discomfort, and anxiety/depression. This brief questionnaire is useful for measuring health status in a wide range of populations and can be immediately utilized for real-world cost-effectiveness analysis between different patient groups [12,14,17,18]. In contrast to the EQ-5D-5L, the EORTC QLQ-C30 is a cancer-specific measure of HRQoL. It consists of five functional scales, nine cancer symptom scales, and a global health/HRQoL scale [15]. The EORTC QLQ-C30 is more detailed compared to the EQ-5D-5L and focuses on specific domains that are relevant from the cancer patient’s perspective. Therefore, clinicians receive more detailed information about the HRQoL of their cancer patients that may not be covered by more generic questionnaires [12,15]. However, before EORTC QLQ-C30 scores can be used for cost-effectiveness analysis, a transformation of data is necessary [19,20,21,22].

For utilizing PROMs in research and clinical care, internal responsiveness is key. Internal responsiveness refers to the ability of a questionnaire to detect changes in a patient’s health status/HRQoL over time (including the changes due to received treatments). Internal responsiveness is crucial for informing clinical decisions and, to a lesser extent, enhancing quality management [23,24,25]. While the internal responsiveness of the EQ-5D-5L and the EORTC QLQ-C30 has been explored in various populations and countries, there remains a significant gap in studies focusing on Dutch breast cancer patients [26,27,28,29,30,31,32,33,34,35,36]. Given that both PROMs are widely used in the Netherlands and the fact that internal responsiveness can vary based on the type of disease, treatment regime, and geographical factors, this study specifically assesses and compares their internal responsiveness among Dutch breast cancer patients during the first year post-surgery [16,23,34]. The findings of this study will offer valuable insights, potentially informing adjustments in research approaches and patient care for breast cancer management in the Netherlands.

## 2. Materials and Methods

### 2.1. Data-Collection

All women diagnosed with invasive breast cancer who underwent surgical treatment with a curative intention at the Erasmus MC Cancer Institute in the Netherlands between 1 November 2015, and 1 January 2022 and who completed EQ-5D-5L and EORTC QLQ-C30 questionnaires pre-operatively (T0, baseline), 6 months post-surgery (T6), and 1-year post-surgery (T12) in the “patient data platform”, Erasmus MC’s online PROM collection tool, were included [16]. Patients who underwent proton therapy or palliative treatment, patients with recurrent breast cancer, and patients without treatment data were excluded. The Institutional Review Board was consulted and concluded that informed consent was not needed since the value-based healthcare strategy is considered the standard of care in Erasmus MC [16].

### 2.2. Surgical Treatment

Surgical interventions of the breast included breast-conserving surgery (BCS) or a mastectomy with or without a reconstruction. Additional chemotherapy, hormonal therapy, and/or (loco)regional radiotherapy were administered to patients following the Dutch national breast cancer guidelines [37,38].

### 2.3. Health Status/HRQoL Assessment

Health status/HRQoL was evaluated with the Dutch EQ-5D-5L (5-level version) and the EORTC QLQ-C30 (version 3.0) at T0, T6, and T12.

The EQ-5D-5L consists of two items: a descriptive system and the EQ visual analog scale (EQ VAS). Each dimension of the descriptive system (mobility, self-care, usual activities, pain/discomfort, and anxiety/depression) has five answer levels, ranging from no problems to extreme problems. The answers to the EQ-5D-5L questions can be transformed into a utility index using the Dutch value set. The EQ-5D-5L index values typically range from 0 to 1, with 0 representing death and 1 representing perfect health. Scores below 0 can occur, indicating that the person feels worse than being dead. The EQ VAS is used to rate the patient’s overall health on a scale of 0–100, with 0 being the best imaginable health and 100 being the worst imaginable health. The empirical reliability and validity of the EQ-5D-5L are documented in previously published literature [14,17,39].

The EORTC QLQ-C30 consists of five functional scales (physical, role, cognitive, emotional, and social), eight cancer symptom scales (fatigue, pain, nausea and vomiting, dyspnea, insomnia, appetite loss, constipation, and diarrhea), a global health/HRQoL scale, and a financial difficulties scale [15]. After linear transformation, all scales range in score from 0–100. For functional/global HRQoL scales, higher scores indicate better functioning; for symptom scales, higher scores indicate greater symptom severity. The empirical reliability and validity of the EORTC QLQ-C30 have previously been established [15,40,41]. The EORTC QLQ-C30 summary score can be calculated from the mean of 13 scales from the EORTC QLQ-C30, excluding the Global Quality of Life scale and the Financial Impact scale. Before determining the mean, symptom scales are reversed to ensure a consistent direction for all scales. Therefore, a higher summary score indicates higher HRQoL [42,43].

### 2.4. Statistical Analysis

Descriptive statistics were used to describe patient, tumor, and treatment characteristics. Means, medians, and standard deviations were used to describe the EQ-5D-5L and EORTC QLQ-C30 scores on T0, T6, and T12. The normality of all scores was assessed using the Kolmogorov–Smirnov and Shapiro–Wilk tests. If normally distributed, the parametric paired *t*-test was used to compare the scores between T0 and T6 and between T0 and T12. If not normally distributed, the non-parametric Wilcoxon signed-rank test was used.

Mean and median differences of the EQ-5D-5L and EORTC QLQ-C30 scores between T0 and T6 (delta 1) and between T0 and T12 (delta 2) were calculated to display within-group change. For the EQ-5D-5L scores and functional scales of the EORTC QLQ-C30, negative deltas indicate a decline in health status/HRQoL over time, while positive deltas indicate an increase in health status/HRQoL. For the symptom scales of the EORTC QLQ-C30, negative deltas indicate an improvement in HRQoL over time, while positive deltas indicate a decline in HRQoL. Mean deltas of the EQ-5D-5L index and EORTC QLQ-C30 Global Quality of Life scale were compared to the minimal clinically important difference (MCID) of the EQ-5D-5L index (0.08) and the EORTC QLQ-C30 (5) to determine clinical significance [44,45]. The MCID of the EQ-5D-5L index was based on the study of Pickard et al. as there are no MCID values available for cancer patients using the Dutch value set of the EQ-5D-5L [44].

To evaluate and compare the internal responsiveness of the two PROMs, effect sizes (ES) and standardized response means (SRM) were calculated. The ES was defined as the mean delta (e.g., the change in score from T0 to T6/T12), divided by the standard deviation of the T0 score. The SRM was calculated as the mean delta, divided by the standard deviation of the delta [23,46]. Both the ES and SRM were classified as large (≥0.8), moderate (0.5–0.79), or small (<0.5) based on previously established criteria [47,48,49]. The ES and SRM are standardized indicators of change in health status/HRQoL over time, regardless of the sample size [23,50].

To gain more insights into the internal responsiveness of the EQ-5D-5L and EORTC QLQ-C30 in different patient groups, a subgroup analysis was performed on patients receiving chemotherapy (neoadjuvant, adjuvant, or both) [37]. The internal responsiveness of the EQ-5D-5L and EORTC QLQ-C30 might be greater for patients receiving chemotherapy since chemotherapy has a significant effect on HRQoL [51].

All statistical analyses were performed using R statistical software version 4.2.2, and a two-sided *p*-value of 5% was considered significant [52].

## 3. Results

**Figure 1:**
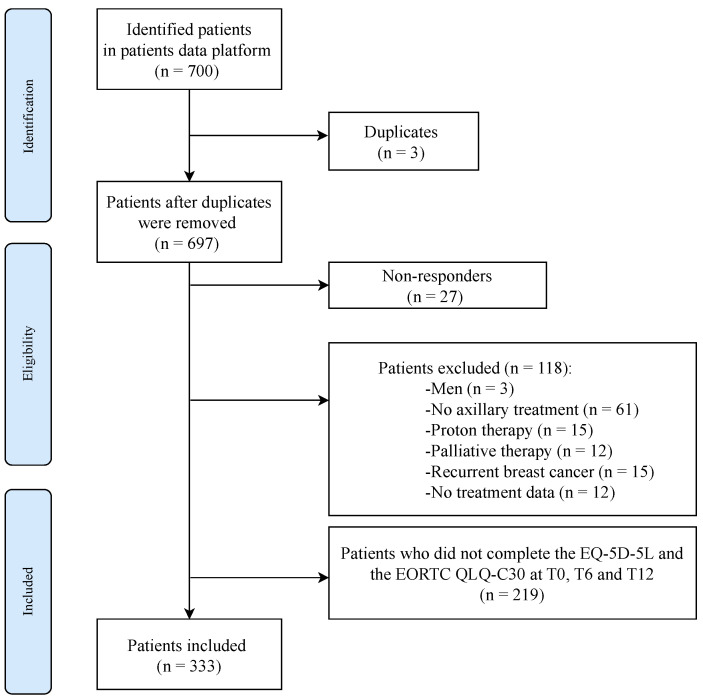
Flowchart. T0, baseline; T6, 6 months post-surgery; T12, 12 months post-surgery.

After removing 3 duplicates and 27 non-responders, 118 patients were further excluded based on the predefined exclusion criteria. Additionally, 219 patients were not included in the analysis due to incomplete responses on the EQ-5D-5L and EORTC QLQ-C30 questionnaires at baseline (T0), 6 months (T6), and 12 months (T12) post-surgery. A total of 333 patients were eligible for the statistical analysis (Figure 1).

### 3.1. Patient Characteristics

The median age of the breast cancer patients was 54 years, ranging from 26.1 to 86.2 years, and 48.3% of the patients had a body mass index (BMI) below 25. Most of the patients were diagnosed with pT1 and pN0 breast cancer and had hormone receptor (HR) positive tumors (67.7%). Breast-conserving surgery (BCS) was the most frequently performed surgical procedure (56.2%). Hormonal therapy was received by 55.3% of the patients, and 41.1% were treated with chemotherapy. The most used axillary treatment was sentinel lymph node biopsy (SLNB) without additional axillary radiotherapy (Table 1).

### 3.2. EQ-5D-5L and EORTC QLQ-C30 Scores over Time

The EQ-5D-5L index score at T6 (*p* < 0.001) and T12 (*p* = 0.006) is significantly lower than that at T0. The Global Quality of Life scale and the summary score of the EORTC QLQ-C30 decrease significantly between T0 and T6 (*p* = 0.010, *p* < 0.001, Table 2). The remaining functional and symptom scales of the EORTC QLQ-C30 tend to show significant differences more frequently between T0 and T6 than between T0 and T12 (Table 2).

### 3.3. Deltas and MCID

**Figure 2:**
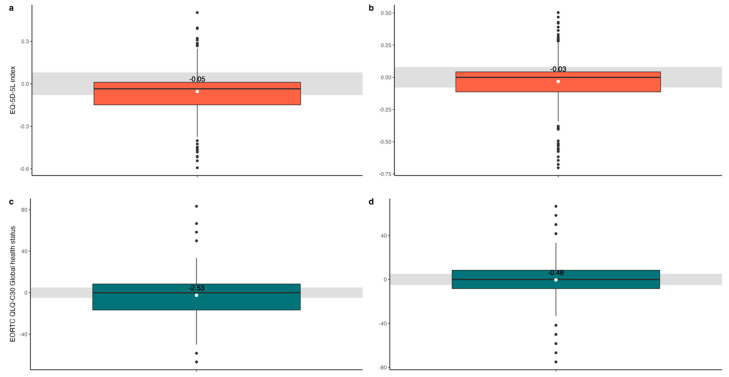
Boxplots of delta 1 (**a**) and delta 2 (**b**) for the EQ-5D-5L index (red) and of delta 1 (**c**) and delta 2 (**d**) for the EORTC QLQ-C30 Global health status (blue). The horizontal line in each boxplot represents the median value, while the white dot indicates the mean. The number within the boxplot represents the mean delta value. The grey interval represents the MCID of the EQ-5D-5L (0.08) and of the EORTC QLQ-C30 (5).

Mean delta 1 (e.g., the difference between T0 and T6) of the EQ-5D-5L index (−0.05) and the EORTC QLQ-C30 Global Quality of Life scale (−2.53) is negative, indicating that health status/HRQoL of patients with breast cancer is lower at T6 compared to T0. In addition, mean delta 2 (e.g., the difference between T0 and T12) of the EQ-5D-5L index (−0.03) and the EORTC QLQ-C30 Global Quality of Life scale (−0.48) is negative, indicating that health status/HRQoL of patients with breast cancer is lower at T12 compared to T0. Delta 1 and 2 of the EQ-5D-5L index and of the EORTC QLQ-C30 Global Quality of Life scale are smaller than the corresponding MCID: (0.08) and (5), respectively (Figure 2 and Appendix A, Table A1). Among the other scales of the EORTC QLQ-C30, only the delta 1 values for the functional scales exceed the MCID (Appendix A, Table A1).

### 3.4. Internal Responsiveness

The ES and SRM for delta 1 and 2 of the EQ-5D-5L and the EORTC QLQ-C30 are all <0.5 (Table 3). Moreover, the ES and SRM tend to be greater for delta 1 compared to delta 2. In addition, the ES and SRM for the EQ-5D-5L index are greater compared to the ES and SRM for the Global Quality of Life scale and summary score of the EORTC QLQ-C30 (Table 3).

### 3.5. Subgroup Analysis

For patients receiving chemotherapy, the ES and SRM for delta 1 and 2 of the EQ-5D-5L and the EORTC QLQ-C30 are generally below 0.5, except for cognitive functioning, pain, and appetite loss (EORTC QLQ-C30). The ES and SRM for delta 1 tend to be greater than those for delta 2 (Table 4). The ES and SRM for delta 1 tend to be greater for patients receiving chemotherapy compared to patients without chemotherapy. Delta 2 does not exhibit a consistent pattern regarding the ES and SRM between the two groups (Table 4 and Appendix A, Table A2).

## 4. Discussion

This study aimed to evaluate and compare the internal responsiveness of the EQ-5D-5L and the EORTC QLQ-C30 among Dutch breast cancer patients during the first year post-surgery. The results revealed that the difference between T0 and T6/T12 of the EQ-5D-5L index value and most of the EORTC QLQ-C30 scales were lower than their respective MCIDs, suggesting that the changes in health status/HRQoL as captured by these PROMs may not be clinically meaningful for the patient. It is important to note that the range of reported MCID values for the EQ-5D and EORTC QLQ-C30 varies widely. This indicates that any conclusions regarding the clinical significance of these changes depend significantly on the chosen MCID threshold. The thresholds used in this study are relatively high, which makes clinically significant changes less likely to be observed [53,54,55,56,57].

Both the EQ-5D-5L and the EORTC QLQ-C30 have relatively small internal responsiveness (<0.5), measured by the ES and SRM, in Dutch breast cancer patients 6 and 12 months after surgery. Interestingly, the EQ-5D-5L index showed slightly greater internal responsiveness compared to the EORTC QLQ-C30 Global Quality of Life scale and summary score. Additionally, the results demonstrated that the internal responsiveness is greater for the period from baseline to 6 months (delta 1) than from baseline to 12 months post-surgery (delta 2), which aligns with previous research demonstrating the decreasing effect of diagnosis and treatment on health status/HRQoL over time and the tendency for health status/HRQoL to return to baseline levels [58,59].

The relatively low breast cancer stage of the patients included in this cohort may have contributed to the small changes in HRQOL/health status. Patients with more advanced breast cancer tend to experience a greater impact on their health status/HRQoL and therefore the internal responsiveness among advanced breast cancer patients may be greater than the internal responsiveness of both PROMs in the current patient cohort [60,61,62]. This is further supported by the subgroup analysis, which demonstrated that the internal responsiveness was slightly greater in patients receiving chemotherapy compared to patients without chemotherapy potentially due to the significant impact of chemotherapy on health status/HRQoL [51]. Patients receiving chemotherapy showed greater internal responsiveness for delta 1 compared to delta 2. This can be attributed to the fact that 6 months post-surgery, patients may still be undergoing chemotherapy and therefore experience chemotherapy-related symptoms, while at 12 months post-surgery, most patients have completed their chemotherapy treatment [37].

Overall, the internal responsiveness of both PROMs was small. However, greater responsiveness was observed at the expected time points and within the specific subgroups. The results of this study are consistent with some previous studies that have examined internal responsiveness in different study populations [26,27,28,29,30,31,32,33,34]. The study by Rundgren et al. found that the internal responsiveness of the EQ-5D was greater between baseline and 3- months post-surgery compared to the period between 3- and 12-months post-surgery in patients with a distal radius fracture [29]. Uwer et al. found that the EORTC QLQ-C30 was more responsive in colorectal cancer patients who received chemotherapy compared to patients who received radiotherapy and concluded that the internal responsiveness of the EORTC QLQ-C30 depends on the type of treatment given [28]. Previous studies that have been conducted in breast cancer patients demonstrated larger responsiveness of the EQ-5D and the EORTC QLQ-C30. It is important to note that these studies had a shorter follow-up period, included patients receiving advanced treatments, or did not analyze the internal responsiveness with the ES and/or SRM [31,32,33]. The study by Kimman et al. examined the internal responsiveness of the EQ-5D with the SRM in breast cancer patients during the first year after treatment. The study found that the EQ-5D was overall responsive, but not responsive enough to detect small changes in health status [30].

Given the variability in findings, where some studies report large internal responsiveness and others report small to moderate, it is evident that the internal responsiveness of health status/HRQoL instruments is influenced by factors such as the length of follow-up, the study population, and the type of intervention [26,27,28,29,30,31,32,33,34]. This underscores the necessity of conducting targeted research on internal responsiveness within specific disease cohorts to tailor insights more accurately. Furthermore, results from such studies must be interpreted with caution, considering the diverse factors that can influence outcomes.

### 4.1. Strengths and Limitations

To our knowledge, this is the first study assessing and comparing the internal responsiveness of the EQ-5D-5L and EORTC QLQ-C30 in Dutch breast cancer patients. As both PROMs are widely used in breast cancer research and clinical practice, the findings of this study provide crucial guidance for refining research methodologies and enhancing patient care strategies in the management of breast cancer [23,24,25]. Another key strength of this study is the availability of a relatively large sample size (more than 300 patients) and the longitudinal nature of the health status/HRQoL data. This is of significance as it is known that the health status/HRQoL of breast cancer patients is dynamic and may change over time [63]. Furthermore, a subgroup analysis was performed on patients undergoing chemotherapy [37]. This subgroup analysis provides insights into the internal responsiveness of both the EQ-5D-5L and EORTC QLQ-C30 in different patient groups. Moreover, given that MCIDs for the EQ-5D-5L and EORTC QLQ-C30 in breast cancer are not well defined, this study provides valuable data for future research to create more robust MCID guidelines [57]. Lastly, the results from this study could inform more accurate sample size calculations for future clinical trials in Dutch breast cancer patients that use these specific PROMs as endpoints.

One limitation of the current study is that only internal responsiveness was examined, which is one of a few measures to consider when selecting a particular PROM. The external responsiveness of the EQ-5D-5L and the EORTC QLQ-C30, which evaluates the degree to which changes in these PROMs correspond to changes in a reference measure of health status/HRQoL, was not assessed due to the absence of a gold standard for assessing health status/HRQoL. The external responsiveness of a PROM is important for clinical practice as this indicates whether the PROM correctly reflects the actual change in the HRQoL of patients [23]. Another limitation to consider is the interaction between the internal responsiveness of PROMs and the actual changes in a patient’s health status/HRQoL over time. When the actual changes in the patient’s health status/HRQoL are minimal, it has an impact on the responsiveness of the PROMs. This is particularly evident in breast cancer patients who maintain the highest stability in their health status/HRQoL compared to patients with other types of cancer [35]. This stability suggests that while PROMs are valuable, their responsiveness may be influenced by the inherent steadiness of health status/HRQoL in certain patient groups, necessitating careful interpretation of results in these contexts. Furthermore, the current study focused solely on the internal responsiveness of the EORTC QLQ-C30 and EQ-5D-5L. In a future study, it might be valuable to also examine and compare the internal responsiveness of commonly used breast cancer-specific PROMs such as the BREAST-Q and EORTC QLQ-BR23. The results will provide a better understanding of the internal responsiveness across a broader spectrum of PROMs and will improve the interpretation of those PROMs in clinical practice. Lastly, the subgroup analysis focused exclusively on chemotherapy while health status and HRQoL might also change depending on other patient and treatment characteristics or the baseline health status/HRQoL score of an individual. For instance, patients with initially low health status/HRQoL scores at baseline may have minimal changes in their health status/HRQoL following surgery, while patients with higher health status/HRQoL scores before surgery might experience significant reductions in their health status/HRQoL post-surgery. However, from a recent study, it is known that age, gender, and baseline HRQoL may not significantly impact the temporal stability of HRQoL measured with the EORTC QLQ-C30 in cancer patients [35].

### 4.2. Clinical Implications

The results of this study indicate that the internal responsiveness of the EQ-5D-5L and EORTC QLQ-C30 in breast cancer patients 6- and 12 months post-surgery is relatively small. Additional approaches to measuring health status/HRQoL in this specific patient cohort may be considered, such as administering PROMs at different or more time points to detect smaller changes in health status/HRQoL and using breast cancer-specific PROMs as recommended by the ICHOM Breast Cancer standard set to improve patient-centered healthcare and research in the future [35,36,64].

## 5. Conclusions

The results indicated that the observed mean differences between baseline and 6- and 12-months post-surgery of the EQ-5D-5L index and most of the EORTC QLQ-C30 scales were below their respective MCIDs at both time points. Internal responsiveness for the EQ-5D-5L and EORTC QLQ-C30 was small (<0.5), with greater responsiveness observed at 6 months compared to 12 months post-surgery. Additionally, the EQ-5D-5L index showed greater internal responsiveness than the Global Quality of Life scale and summary score of the EORTC QLQ-C30. These findings provide crucial insights for interpreting outcomes from both PROMs in Dutch breast cancer research and clinical practice.

## Figures and Tables

**Table 1:** Baseline characteristics of the study population.

Characteristics	Patients (*n* = 333)
*Age*	
Mean (SD)	54.0 (13.9)
Median [Min, Max]	53.9 [26.1, 86.2]
*BMI category*	
<25	161 (48.3%)
25–30	122 (36.6%)
>30	50 (15.0%)
*Type of breast surgery*	
Mastectomy	91 (27.3%)
BCS	187 (56.2%)
Mastectomy + reconstruction	55 (16.5%)
*Receptor status*	
Triple negative	47 (14.1%)
HER2 positive	38 (11.4%)
HR positive and HER2 negative	225 (67.6%)
Unknown	23 (6.9%)
*Primary tumor stage*	
pT0	29 (8.7%)
pT1	187 (56.2%)
pT2	69 (20.7%)
pT3	9 (2.7%)
pT4	0 (0%)
pTis	33 (9.9%)
pTmi	6 (1.8%)
*Regional lymph nodes stage*	
pN0	249 (74.8%)
pN1	66 (19.8%)
pN2	14 (4.2%)
pN3	4 (1.2%)
*Hormonal therapy*	
No	149 (44.7%)
Yes	184 (55.3%)
*Chemoimmunotherapy*	
No	196 (58.9%)
Yes	137 (41.1%)
*Axillary treatment*	
SLNB/RISAS	231 (69.4%)
ALND	35 (10.5%)
SLNB/RISAS + Rtx	27 (8.1%)
ALND + Rtx	40 (12.0%)

Abbreviations: ALND, axillary lymph node dissection; BCS, breast-conserving surgery; BMI, body mass index; HR, hormone receptor; RISAS, radioactive iodine seed localization in the axilla with sentinel node procedure; Rtx, radiotherapy; SLNB, sentinel lymph node biopsy. Note: HER2 positive, if HER2 is positive; HR-positive, estrogen receptor+ and/or progesterone receptor+ and HER2−; triple-negative, estrogen receptor- and progesterone receptor- and HER2−.

**Table 2:** EQ-5D-5L and EORTC QLQ-C30 scores pre-operatively, at 6- and 12 months post-surgery.

	T0	T6	T12	*p*-Value (vs. T0)	
HRQoL	(*n* = 333)	(*n* = 333)	(*n* = 333)	T6	T12
*EQ-5D-5L*					
*Index*					
Mean (SD)	0.852 (0.143)	0.799 (0.170)	0.820 (0.192)	**<0.001**	**0.006**
Median [Min, Max]	0.883 [0.271, 1.00]	0.818 [−0.182, 1.00]	0.852 [−0.261, 1.00]		
*VAS score*					
Mean (SD)	78.0 (16.4)	74.3 (15.7)	76.1 (15.4)	**<0.001**	**0.039**
Median [Min, Max]	80.0 [5.00, 100]	78.0 [8.00, 100]	80.0 [2.00, 100]		
*EORTC QLQ-C30*					
*Global Quality of Life scale*					
Mean (SD)	77.6 (17.3)	75.1 (18.4)	77.1 (18.2)	**0.010**	0.783
Median [Min, Max]	83.3 [16.7, 100]	83.3 [0, 100]	83.3 [0, 100]		
*Emotional functioning*					
Mean (SD)	74.5 (21.1)	78.9 (22.7)	82.4 (20.1)	**<0.001**	**<0.001**
Median [Min, Max]	83.3 [0, 100]	83.3 [0, 100]	91.7 [0, 100]		
*Role functioning*					
Mean (SD)	83.9 (24.3)	74.6 (27.1)	79.7 (26.6)	**<0.001**	**0.007**
Median [Min, Max]	100 [0, 100]	83.3 [0, 100]	83.3 [0, 100]		
*Physical functioning*					
Mean (SD)	90.1 (15.4)	84.5 (16.4)	86.9 (15.2)	**<0.001**	**<0.001**
Median [Min, Max]	93.3 [20.0, 100]	86.7 [26.7, 100]	93.3 [20.0, 100]		
*Cognitive functioning*					
Mean (SD)	87.7 (16.4)	80.0 (23.4)	82.8 (20.5)	**<0.001**	**<0.001**
Median [Min, Max]	100 [16.7, 100]	83.3 [0, 100]	83.3 [0, 100]		
*Social functioning*					
Mean (SD)	88.5 (20.2)	81.7 (25.1)	85.8 (22.2)	**<0.001**	0.122
Median [Min, Max]	100 [0, 100]	100 [0, 100]	100 [0, 100]		
*Fatigue*					
Mean (SD)	22.9 (21.8)	31.8 (24.1)	25.4 (22.4)	**<0.001**	0.076
Median [Min, Max]	22.2 [0, 100]	22.2 [0, 100]	22.2 [0, 100]		
*Nausea and vomiting*					
Mean (SD)	4.96 (12.3)	4.71 (11.0)	3.65 (10.1)	0.709	0.187
Median [Min, Max]	0 [0, 83.3]	0 [0, 83.3]	0 [0, 100]		
*Pain*					
Mean (SD)	13.5 (19.0)	20.9 (22.1)	19.6 (21.7)	**<0.001**	**<0.001**
Median [Min, Max]	0 [0, 83.3]	16.7 [0, 100]	16.7 [0, 100]		
*Dyspnea*					
Mean (SD)	7.41 (16.9)	13.9 (23.6)	9.91 (19.1)	**<0.001**	**0.014**
Median [Min, Max]	0 [0, 100]	0 [0, 100]	0 [0, 100]		
*Insomnia*					
Mean (SD)	27.7 (27.7)	28.6 (30.0)	25.8 (28.2)	0.325	0.589
Median [Min, Max]	33.3 [0, 100]	33.3 [0, 100]	33.3 [0, 100]		
*Appetite loss*					
Mean (SD)	12.4 (21.8)	7.61 (18.2)	4.70 (13.7)	**<0.001**	**<0.001**
Median [Min, Max]	0 [0, 100]	0 [0, 100]	0 [0, 100]		
*Constipation*					
Mean (SD)	7.41 (18.1)	8.51 (20.0)	8.21 (18.8)	0.334	0.279
Median [Min, Max]	0 [0, 100]	0 [0, 100]	0 [0, 100]		
*Diarrhea*					
Mean (SD)	5.91 (15.1)	4.10 (13.2)	3.80 (12.9)	0.134	0.099
Median [Min, Max]	0 [0, 100]	0 [0, 66.7]	0 [0, 66.7]		
*Financial difficulties*					
Mean (SD)	4.10 (14.6)	8.61 (21.2)	6.91 (18.7)	**<0.001**	**0.01**
Median [Min, Max]	0 [0, 100]	0 [0, 100]	0 [0, 100]		
*Summary score*					
Mean (SD)	86.3 (11.4)	83.0 (13.5)	85.9 (12.5)	**<0.001**	0.746
Median [Min, Max]	90.1 [32.6, 100]	85.4 [27.9, 100]	88.8 [28.6, 100]		

Abbreviations: T0, baseline; T6, 6 months post-surgery; T12, 12 months post-surgery. Note: EORTC QLQ-C30 functional/global HRQoL scales, higher scores indicate better functioning; symptom scales, higher scores indicate greater symptom severity. Bold indicates *p* < 0.05.

**Table 3:** ES and SRM of delta 1 and delta 2.

HRQoL	SRM: Delta 1	SRM: Delta 2	ES: Delta 1	ES: Delta 2
*EQ-5D-5L*				
Index	−0.339	−0.176	−0.374	−0.224
VAS score	−0.199	−0.104	−0.224	−0.115
*EORTC QLQ-C30*				
Global Quality of Life scale	−0.133	−0.024	−0.146	−0.028
Physical functioning	−0.388	−0.230	−0.366	−0.205
Role functioning	−0.322	−0.146	−0.386	−0.175
Emotional functioning	0.182	0.331	0.207	0.371
Cognitive functioning	−0.352	−0.231	−0.468	−0.297
Social functioning	−0.273	−0.11	−0.337	−0.131
Fatigue	0.354	0.109	0.407	0.115
Nausea and vomiting	−0.017	−0.093	−0.020	−0.106
Pain	0.295	0.261	0.389	0.321
Dyspnea	0.268	0.126	0.385	0.148
Insomnia	0.029	−0.059	0.033	−0.069
Appetite loss	−0.194	−0.340	−0.221	−0.354
Constipation	0.049	0.037	0.061	0.044
Diarrhea	−0.099	−0.119	−0.119	−0.139
Financial difficulties	0.222	0.145	0.308	0.192
Summary score	−0.255	−0.036	−0.290	−0.040

Abbreviations: SRM, standardized response mean; ES, effect size. Note: delta 1, difference in scores between T0 and T6; delta 2, difference in scores between T0 and T12.

**Table 4:** Subgroup analysis: ES and SRM of delta 1 and delta 2 in patients receiving chemotherapy.

HRQoL	SRM: Delta 1	SRM: Delta 2	ES: Delta 1	ES: Delta 2
*EQ-5D-5L*				
Index	−0.331	−0.141	−0.394	−0.172
VAS score	−0.221	−0.050	−0.247	−0.049
*EORTC QLQ-C30*				
Global Quality of Life scale	−0.260	0.013	−0.296	0.013
Physical functioning	−0.436	−0.149	−0.399	−0.140
Role functioning	−0.381	−0.079	−0.452	−0.097
Emotional functioning	0.126	0.300	0.149	0.341
Cognitive functioning	−0.403	−0.291	−**0.563**	−0.373
Social functioning	−0.298	−0.083	−0.386	−0.095
Fatigue	0.380	0.055	0.435	0.058
Nausea and vomiting	−0.042	−0.125	−0.053	−0.141
Pain	0.351	0.237	**0.501**	0.293
Dyspnea	0.294	0	0.475	0
Insomnia	0.008	−0.112	0.009	−0.139
Appetite loss	−0.334	−**0.505**	−0.41	−**0.540**
Constipation	−0.021	0	−0.022	0
Diarrhea	−0.156	−0.209	−0.198	−0.255
Financial difficulties	0.308	0.233	0.465	0.310
Summary score	−0.236	0.060	−0.282	0.067

Abbreviations: SRM, standardized response mean; ES, effect size. Note: delta 1, difference in scores between T0 and T6; delta 2, difference in scores between T0 and T12. Bold indicates SRM/ES > 0.5.

## Data Availability

The datasets generated during and/or analyzed during the current study are not publicly available due to privacy or ethical restrictions but are available from the corresponding author on reasonable request.

## Appendix A

**Table A1:** Deltas EQ-5D-5L and EORTC QLQ-C30.

HRQoL	Delta 1 (*n* = 333)	Delta 2 (*n* = 333)
*EQ-5D-5L*		
*Index*		
Mean (SD)	−0.054 (0.158)	−0.032 (0.182)
Median [Min, Max]	−0.035 [−0.593, 0.503]	0 [−0.702, 0.503]
*VAS score*		
Mean (SD)	−3.69 (18.5)	−1.89 (18.2)
Median [Min, Max]	−1.00 [−69.0, 54.0]	0 [−74.0, 55.0]
*EORTC QLQ-C30*		
*Global Quality of Life scale*		
Mean (SD)	−2.53 (18.9)	−0.476 (19.9)
Median [Min, Max]	0 [−66.7, 83.3]	0 [−75.0, 66.7]
*Emotional functioning*		
Mean (SD)	4.36 (23.9)	**7.83 (23.6)**
Median [Min, Max]	8.33 [−75.0, 91.7]	8.33 [−75.0, 83.3]
*Role functioning*		
Mean (SD)	−**9.36 (29.1)**	−4.25 (29.1)
Median [Min, Max]	0 [−100, 100]	0 [−100, 100]
*Physical functioning*		
Mean (SD)	−**5.63 (14.5)**	−3.14 (13.7)
Median [Min, Max]	−6.66 [−66.7, 60.0]	0 [−66.7, 66.7]
*Cognitive functioning*		
Mean (SD)	−**7.66 (21.8)**	−4.85 (21.0)
Median [Min, Max]	0 [−100, 66.7]	0 [−83.3, 66.7]
*Social functioning*		
Mean (SD)	−**6.81 (24.9)**	−2.65 (24.1)
Median [Min, Max]	0 [−100, 100]	0 [−100, 66.7]
*Fatigue*		
Mean (SD)	**8.88 (25.1)**	2.50 (23.0)
Median [Min, Max]	11.1 [−88.9, 88.9]	0 [−77.8, 77.8]
*Nausea and vomiting*		
Mean (SD)	−0.250 (15.1)	−1.30 (13.9)
Median [Min, Max]	0 [−66.7, 83.3]	0 [−83.3, 50.0]
*Pain*		
Mean (SD)	**7.41 (25.1)**	**6.11 (23.4)**
Median [Min, Max]	0 [−83.3, 83.3]	0 [−83.3, 100]
*Dyspnea*		
Mean (SD)	**6.51 (24.3)**	2.50 (19.8)
Median [Min, Max]	0 [−100, 100]	0 [−100, 66.7]
*Insomnia*		
Mean (SD)	0.901 (31.3)	−1.90 (32.3)
Median [Min, Max]	0 [−100, 100]	0 [−100, 100]
*Appetite loss*		
Mean (SD)	−4.80 (24.8)	−**7.71 (22.6)**
Median [Min, Max]	0 [−100, 100]	0 [−100, 66.7]
*Constipation*		
Mean (SD)	1.10 (22.6)	0.801 (21.5)
Median [Min, Max]	0 [−100, 100]	0 [−100, 100]
*Diarrhea*		
Mean (SD)	−1.80 (18.2)	−2.10 (17.7)
Median [Min, Max]	0 [−100, 66.7]	0 [−100, 66.7]
*Financial difficulties*		
Mean (SD)	4.50 (20.3)	2.80 (19.3)
Median [Min, Max]	0 [−100, 100]	0 [−100, 100]
*Summary score*		
Mean (SD)	−3.31 (13.0)	−0.459 (12.7)
Median [Min, Max]	−1.97 [−62.4, 40.9]	0 [−48.0, 48.1]

Note: delta 1, difference in scores between T0 and T6; delta 2, difference in scores between T0 and T12. Bold indicates delta > MCID of the EQ-5D-5L (0.08) or the EORTC QLQ-C30 (5).

**Table A2:** Subgroup analysis: ES and SRM of delta 1 and delta 2 in patients without chemotherapy.

HRQoL	SRM: Delta 1	SRM: Delta 2	ES: Delta 1	ES: Delta 2
*EQ-5D-5L*				
Index	−0.350	−0.200	−0.365	−0.269
VAS score	−0.182	−0.138	−0.208	−0.168
*EORTC QLQ-C30*				
Global Quality of Life scale	−0.032	−0.048	−0.033	−0.059
Physical functioning	−0.351	−0.307	−0.341	−0.258
Role functioning	−0.276	−0.203	−0.343	−0.247
Emotional functioning	0.236	0.360	0.262	0.409
Cognitive functioning	−0.315	−0.181	−0.400	−0.239
Social functioning	−0.258	−0.133	−0.304	−0.169
Fatigue	0.334	0.154	0.401	0.167
Nausea and vomiting	0.006	−0.067	0.008	−0.078
Pain	0.253	0.278	0.313	0.339
Dyspnea	0.253	0.252	0.312	0.269
Insomnia	0.044	−0.017	0.050	−0.019
Appetite loss	−0.040	−0.193	−0.041	−0.194
Constipation	0.101	0.079	0.148	0.091
Diarrhea	−0.045	−0.034	−0.051	−0.038
Financial difficulties	0.140	0.063	0.165	0.083
Summary score	−0.283	−0.124	−0.318	−0.145

Abbreviations: SRM, standardized response mean; ES, effect size. Note: delta 1, difference in scores between T0 and T6; delta 2, difference in scores between T0 and T12.
